# Complementary Supramolecular Functionalization Enhances Antifouling Surfaces: A Ureidopyrimidinone-Functionalized Phosphorylcholine Polymer

**DOI:** 10.1021/acsbiomaterials.3c00425

**Published:** 2023-07-06

**Authors:** Antonio J. Feliciano, Eduardo Soares, Anton W. Bosman, Clemens van Blitterswijk, Lorenzo Moroni, Vanessa L. S. LaPointe, Matthew B. Baker

**Affiliations:** † Maastricht University, MERLN, Universiteitssingel 40, 6229 ER Maastricht, The Netherlands; ‡ SupraPolix B.V., Horsten 1, 5612 AX Eindhoven, The Netherlands

**Keywords:** 2-methacryloyloxyethyl phosphorylcholine, zwitterionic, UPy, supramolecular, coating

## Abstract

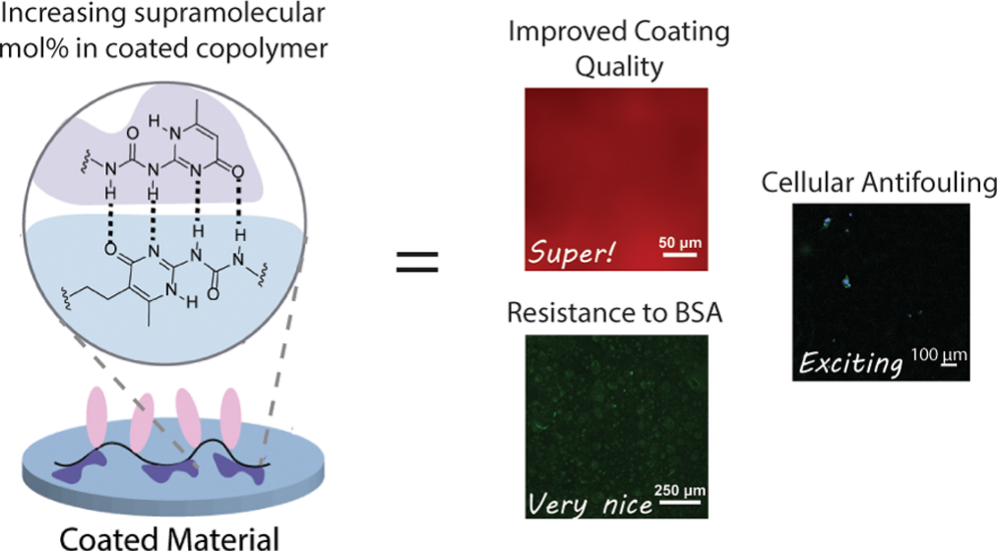

Fibrosis of implants remains a significant challenge in the use of biomedical devices and tissue engineering materials. Antifouling coatings, including synthetic zwitterionic coatings, have been developed to prevent fouling and cell adhesion to several implantable biomaterials. While many of these coatings need covalent attachment, a conceptually simpler approach is to use a spontaneous self-assembly event to anchor the coating to a surface. This could simplify material processing through highly specific molecular recognition. Herein, we investigate the ability to utilize directional supramolecular interactions to anchor an antifouling coating to a polymer surface containing a complementary supramolecular unit. A library of controlled copolymerization of ureidopyrimidinone methacrylate (UPyMA) and 2-methacryloyloxyethyl phosphorylcholine (MPC) was prepared and their UPy composition was assessed. The MPC-UPy copolymers were characterized by ^1^H NMR, Fourier transform infrared (FTIR), and gel permeation chromatography (GPC) and found to exhibit similar mol % of UPy as compared to feed ratios and low dispersities. The copolymers were then coated on an UPy elastomer and the surfaces were assessed for hydrophilicity, protein absorption, and cell adhesion. By challenging the coatings, we found that the antifouling properties of the MPC-UPy copolymers with more UPy mol % lasted longer than the MPC homopolymer or low UPy mol % copolymers. As a result, the bioantifouling nature could be tuned to exhibit spatio-temporal control, namely, the longevity of a coating increased with UPy composition. In addition, these coatings showed nontoxicity and biocompatibility, indicating their potential use in biomaterials as antifouling coatings. Surface modification employing supramolecular interactions provided an approach that merges the simplicity and scalability of nonspecific coating methodology with the specific anchoring capacity found when using conventional covalent grafting with longevity that could be engineered by the supramolecular composition itself.

## 1 Introduction

As more medical devices are developed and implanted in the body, the need for functional surfaces that prevent biomolecule and cellular adhesion is only increasing. For many implants, the foreign-body response is a known complication and devices coated with antifouling polymers have been developed to reduce fibrosis in vivo.^1^ Poly(ethylene glycol), a neutral and hydrophilic polymer, has been widely studied for its biocompatibility and antifouling properties.^2,3^ However, alternatives to PEG are needed as it can be oxidized, patients can become sensitized over time, and the antifouling ability is imperfect.^4^ Zwitterionic materials, such as those based on sulfobetaines (SBs)^5^ and phosphorylcholines (PCs),^6^ are common alternatives and have been employed in many formulations for different medical device coatings such as stents, biosensors, and drug carriers.^7−9^ These hydrophilic polymers have shown ultralow fouling even when in contact with undiluted human plasma or serum.^10^

Techniques to attach antifouling coatings to device surfaces include electrostatic interactions, surface grafting, and, more recently, supramolecular or physical interactions.^11^ Self-assembled monolayers (SAMs) utilize positively and negatively charged electrostatic attraction for surface adhesion; however, thicker coatings are necessary to stabilize them.^12^ In dental applications, copolymers of 2-methacryloyloxyethyl phosphorylcholine (MPC) and Ca^2+^-binding moieties were synthesized to bind with hydroxyapatite to prevent fouling and bacterial adhesion.^13^ “Grafting-from” zwitterionic brushes have also been extensively explored; for instance, Kuang and Messersmith used catecholamine peptide ATRP initiators to graft poly(sulfobetaine methacrylate) (SBA) to Au, TiO_2_, SiO_2_, and polycarbonate.^14^ Alternatively, in a “grafting-to” approach, researchers covalently functionalized alginate hydrogels with thiol-containing MPC copolymers. After implanting the hydrogels subcutaneously in mice for two weeks, they found that their copolymer coating reduced the fibrotic response.^15^ While these methods of attachment impart excellent antifouling properties, they may not be compatible with every biomaterial or application. Tricky reaction conditions (surface treatment, salinization, free radicals) in addition to lengthy purifications limit the types of materials that can be successfully employed.^16^

Supramolecular adhesion from host–guest, hydrogen-bonding, and metal–ligand interactions is an interesting coating approach due to their noncovalent nature, modularity, and biomimetic interaction.^17^ The convenience of supramolecular recognition at an interface has led to the design of supramolecular switches, reversibility in absorption and fouling, self-healing, and drug release.^18−21^ The spontaneous nature of the noncovalent bond in many supramolecular materials enables the formation and functionalization under mild and straightforward conditions and can allow for temporal control of the interaction. Ureidopyrimidinone (UPy), a self-complementary hydrogen-bonding supramolecular moiety, has been used as a monomer unit to impart strength and processability to biomaterials and has already found their way to the clinic.^22−26^ With a strong association constant, *K*_dim_ = 6 × 10^8^ M^–1^ in chloroform, UPy has been explored as an adhesive or supramolecular anchoring unit in different systems.^27^ Side-chain polymers, such as methacrylate copolymers, were found to increase in stiffness and peel strength with increasing UPy concentration.^28^ Additionally, hydrogels have been made from copolymers with hydroxyethyl methacrylate (HEMA) and UPyMA, where gelation occurred through self-dimerization between polymer chains.^29,30^

To control the fouling properties of polymers with the supramolecular UPy motif, researchers have made great strides with PEG. Taking advantage of the UPy’s modularity, a mix and match approach has been used to prevent cell adhesion on polycaprolactone vascular grafts, where in vitro studies showed a decrease in cellular adhesion after 24 h and reduced cell infiltration in vivo.^31^ Other methods for functionalizing supramolecular elastomers include ATRP, where zwitterionic SBMA was grafted from UPy-PCL^32^ and covalently attaching a coating of UPy-PEG through an inverse Diels–Alder cycloaddition.^33^ Electrospun fibers of UPy-functionalized poly(hexamethylene carbonate) have also been blended with functionalized PEG with UPy for improving drug retention.^34^ However, we were interested if simple supramolecular coatings of UPy-containing materials via molecular recognition would facilitate enhanced antifouling properties.

**Figure 1:**
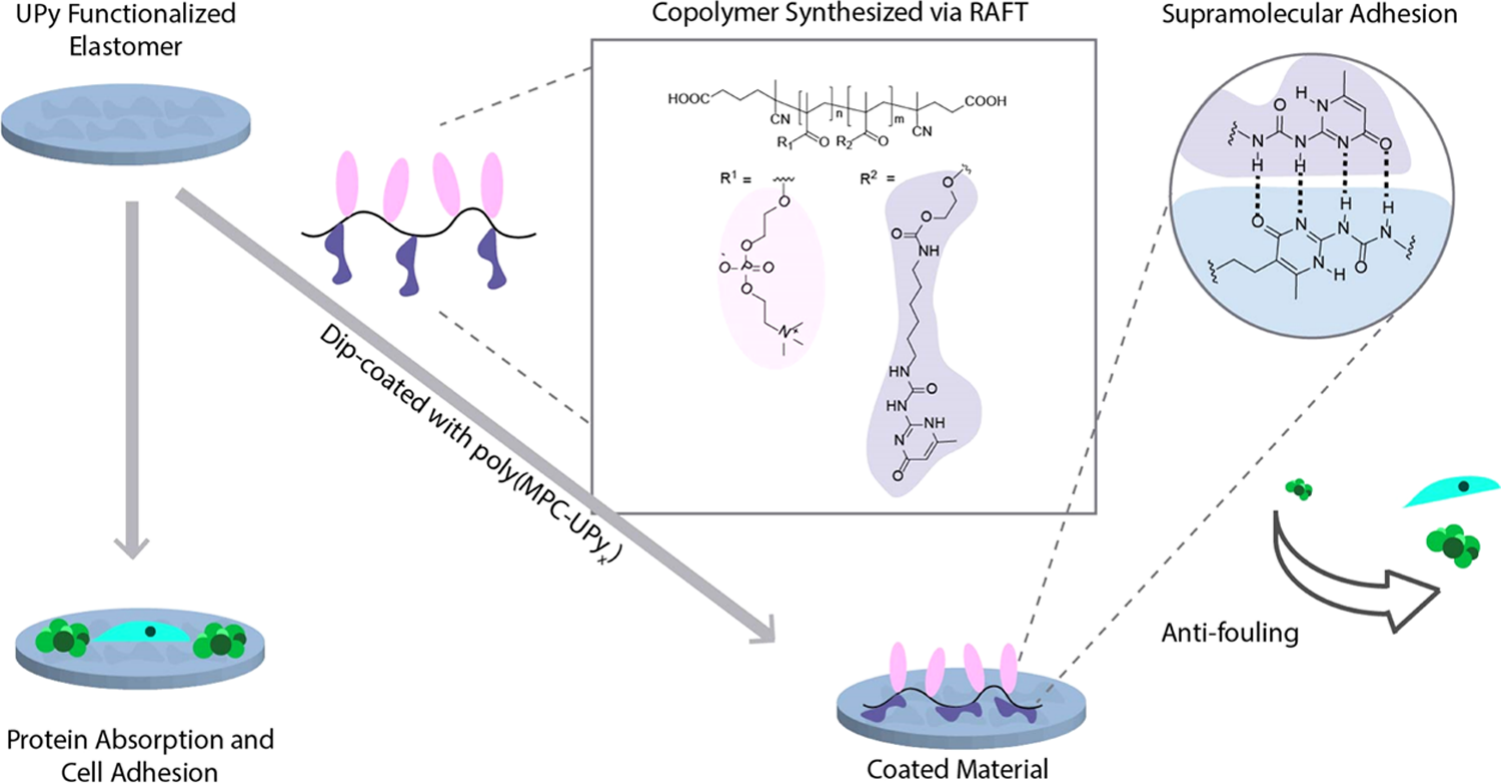
Schematic illustration depicting copolymers anchored to an UPy functionalized elastomer to prevent protein absorption via supramolecular UPy dimerization.

Gallols, phenols, and *n*-butyl acrylates have been copolymerized with MPC to immobilize copolymers in a similar facile dip-coating process,^35^ but in our approach (Figure 1), we designed a UPy-functionalized zwitterionic copolymer that could use directional molecular interactions to facilitate coating adhesion to a UPy-containing biomedical elastomer.^36^ By synthesizing poly(MPC-co-UPyMA), we hypothesized that the MPC groups would present at the surface and act as the antifouling agent while UPy groups act as a tethering group to the UPy elastomeric material. Via reversible addition–fragmentation chain-transfer (RAFT) polymerization, we prepared a library of UPy copolymers with variable UPy concentrations (0–5 mol %), designated as MPC-UPy*_x_*, where *x* is the mol % of UPy in the feedstock. In this study, we characterize the polymers by ^1^H NMR, Fourier transform infrared (FTIR) spectroscopy, gel permeation chromatography (GPC), differential scanning calorimetry (DSC), water contact angle, BSA absorption, toxicity, and cell adhesion as a potential coating material. We found that the UPy-containing antifouling coatings significantly increased coating longevity and decreased cell adhesion when compared to homopolymers of MPC.

## 2 Materials and Methods

### 2.1 Materials

UPy-isocyanate synthon (**1**) and elastomeric chain-extended UPy comprising a poly(hexamethylene carbonate) backbone (UPyE) material^34^ was provided by SupraPolix (Eindhoven, NL). 1,1,1,3,3,3-Hexafluoroisopropanol (HFIP) was purchased from BioSolve. Hydroxyethyl methacrylate (HEMA), chloroform, diethyl ether, dibutyltin dilaurate (DBTDL), 2-(methacryloyloxy)ethyl 2-(trimethylammonio)ethylphosphate (MPC), 4-cyano-4 (phenylcarbonothioylthio)pentanoic acid, 4,4′-azobis 4-cyanovaleric acid (ACVA), chloroform-*d*, methanol-*d*_4_, PBS tablets, methanol, *N*,*N*-dimethylacetamide (DMAc), and rhodamine 6G were purchased from Sigma-Aldrich. Alexa-Fluor-488-labeled BSA and SnakeSkin Dialysis Tubing (10k MWCO) were purchased from Thermo Fisher Scientific. These reagents were used as received and not purified unless otherwise indicated.

### 2.2 Synthesis of UPy Methacrylate (**2**) Monomer

**Scheme 1:**
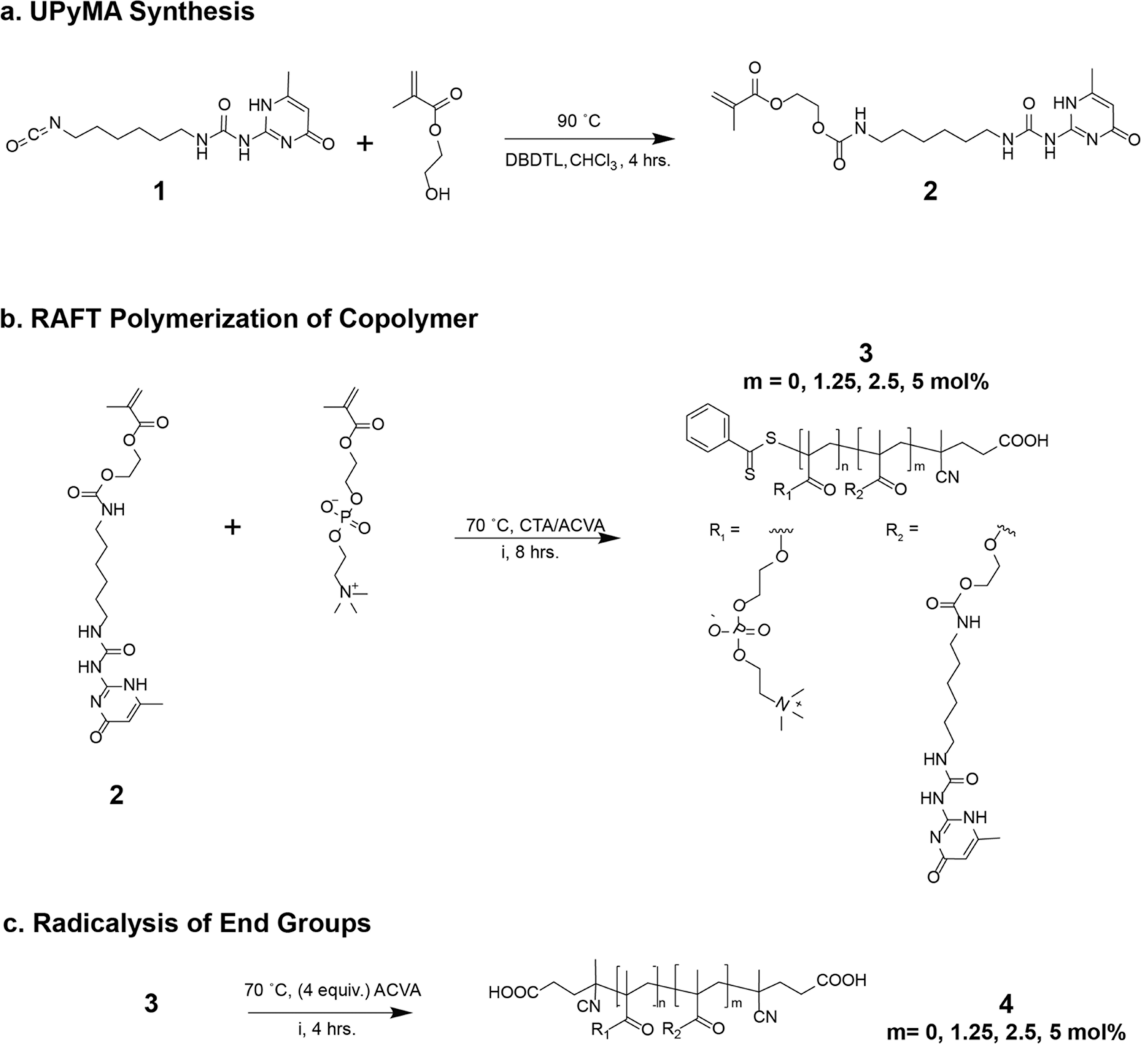
Synthesis of UPyMA Monomer **2** and MPC-UPy*_x_* Copolymer **3** by RAFT Polymerization and Telechelic Diacid **4** by Radicalysis DBDTL: dibutyltin dilaurate, CTA: cyano-4-(phenylcarbonothioylthio)pentanoic acid, ACVA: 4,4′-azobis(4-cyanovaleric acid), and i: methanol/DMAc solvent system.

UPyMA (**2**) was synthesized as previously reported.^27^ In a 25 mL round-bottom flask, iso-UPy synthon (**1**) (0.79 g, 2.69 mmol) was suspended in dry chloroform (15 mL) with HEMA (0.69 g, 5.30 mmol) and DBTDL (8.0 mg, 0.01 mmol) under a nitrogen atmosphere (Scheme 1a). The mixture was stirred on an oil bath for 4 h at 90 °C. The reaction was then cooled to RT. The product precipitated overnight in the fridge and was filtered and washed with excess diethyl ether yielding **2** as a white solid (1.04 g, 91% yield). ^1^H NMR (400 MHz, CDCl_3_): δ 13.1 (s, 1H), 11.9 (s, 1H), 10.1 (t, 1H), 6.1 (d, *J* = 16.2 Hz, 1H), 5.8 (s, 1H), 5.6 (d, *J* = 19.0 Hz, 1H), 5.0 (t, 1H), 4.3 (m, 4H), 3.3 (q, *J* = 12.9, 6.5 Hz, 2H), 3.2 (q, *J* = 13.0, 6.5 Hz, 2H), 2.2 (s, 3H), 2.0 (s, 3H), 1.6–1.2 (m, 8H). Detailed ^1^H NMR spectra in CDCl_3_ can be found in Figure S1 and matches literature precedent.^27^

### 2.3 Synthesis of MPC-UPy_5_ (**3**)

**Figure 2:**
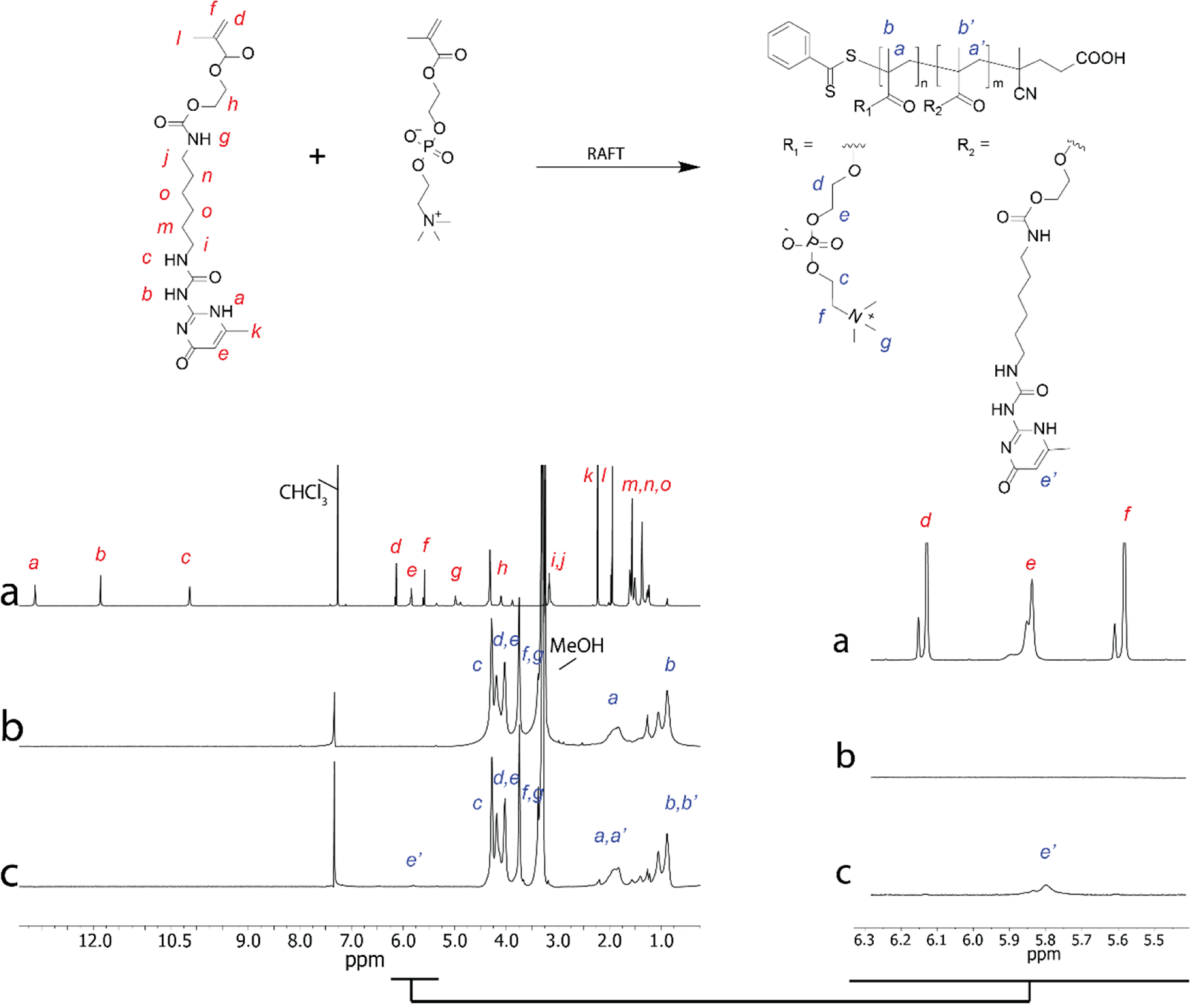
Characterization of UPy-containing polymers determined by ^1^H NMR. Spectra of (a) UPyMA monomer 2 in CDCl_3_. Monomeric protons (red) of 2 include methacrylate protons d and f along with characteristic hydrogen-bonding protons of UPy (a–c). Spectra of (b) purified MPC homopolymer 3 with 0% UPy in CDCl_3_/MeOD and (c) purified copolymer 3 with 5 mol % UPy copolymer in CDCl_3_/MeOD. Polymeric protons (blue) show large presence of MPC protons c–g. UPy’s aromatic singlet proton e’ is present at 5.8 ppm on the copolymer and used to determine the mol % of UPy. Right inset shows magnified view of the spectra between 5.5 and 6.3 ppm.

Utilizing a RAFT polymerization technique, we prepared different UPy mol % copolymers with MPC (Scheme 1). For 5 mol %, UPyMA **2** (0.15 g, 0.35 mmol), MPC monomer (2.0 g, mmol), cyano-4-(phenylcarbonothioylthio)pentanoic acid (10 mg, 0.35 mmol), and 4,4′-azobis(4-cyanovaleric acid) (2.0 mg, 7.1 μmol) were added into a 15 mL single-necked round-bottom flask. Methanol (3.6 mL) and DMAc (2.4 mL) were added as proposed in a similar procedure.^15^ The mixture was stirred with a magnetic stir bar. After sealing the flask with a rubber septum, nitrogen was purged through with an outlet for 10 min, then the outlet was removed and nitrogen allowed to fill the flask and left for an additional 10 min. The reaction was initiated by immersing the flask into an oil bath at 70 °C, Scheme 1b. Polymerization was terminated by cooling to RT and exposure to air after 8 h. The polymer was purified by dialysis (10 K MWCO) in water for two days and the dialysate changed once per day, then lyophilized to yield a light pink solid. To remove residual UPyMA monomer, the lyophilized polymer was washed with warm chloroform. The yield at 8 h (51% conversion) was 44% (0.95 g). Chemical structures and *M*_n_ were determined by ^1^H NMR (Bruker 400) and can be found in Table 1 and Figure S2. Monomer conversion was confirmed by the acrylate vinyl peaks at 6.15 and 5.59 ppm of the crude mixture while the UPy mol % was calculated by UPy’s aromatic singlet proton peak at 5.8 ppm of the purified product labeled e’ in Figure 2. Size exclusion chromatography was also used to assess *M*_n_ and traces can be found in Figure S4.

**Table 1:** Characteristics of Synthesized Polymers

				^1^H NMR			GPC		DSC
polymer	UPy feed (mol %)	*M*_n_^Th^ (g/mol)	conversion at 8 h (%)	*M*_n_^a^	UPy (mol %)	UPy/chain (g/mol)	*M*_n_^b^	*Đ*	*T*_g_ (°C)
MPC	0	55 876	54	35 000	0	0	60 000 (21 000^c^ in H_2_O)	1.1	25
MPC-UPy_1.25_	1.25	56 399	63	37 000	0.4	1.0	59 000	1.3	15
MPC-UPy_2.5_	2.5	56 923	51	31 000	1.0	1.0	25 000	1.2	7
MPC-UPy_5_	5	57 971	52	33 000	4.0	4.0	25 000	1.2	21

a Calculated by end-group analysis of **3** in ^1^H NMR.

b GPC (Mobile phase: HFIP).

c GPC (Mobile phase: aqueous).

### 2.4 Radicalysis of MPC-UPy_5_ (**3**)

Removal of the aromatic CTA was performed by a radicalysis method to synthesize the carboxylic acid homotelechelic polymer (**4**). Typically, purified copolymer **1** (0.95 g) and a fourfold excess of free radical initiator ACVA (0.05 g, 0.178 mmol, 4 equiv to starting CTA in **3**) was weighed and transferred into a 10 mL Schlenk flask. Methanol (3.6 mL) and DMAc (2.4 mL) were added and mixed to solubilize the solids. After sealing the flask, nitrogen was purged for 10 min to the reaction vessel to create an inert atmosphere. The reaction was allowed to mix for 4 h at 70 °C, Scheme 1c. To separate the polymer from the cleaved CTA, we cooled and dialyzed (10 K MWCO) the mixture against water for two days with changes of the dialysate twice (once per day). The resulting polymer (**4**) lost its pink color and was a white solid. Yield was typically around 80% (0.76 g). Removal of CTA from the polymer backbone was confirmed by the loss of aromatic protons 7.2–7.8 ppm of the purified material. ^1^H NMR (Bruker 400) was used to confirm the loss of CTA and size exclusion chromatography was also used to assess M_n_ and detailed in Figure S4. Library of polymer NMR’s post radicalysis can be found in Figure S5.

### 2.5 NMR

For small molecules (**1** and **2**), 1–2 mg of sample was mixed with 600 μL of chloroform-*d*. For copolymers, a mixture of 90:10 chloroform-*d* and methanol-*d*_4_, respectively, was used to solubilize the material and record NMR spectra. NMR spectra were recorded by a Bruker Avance III HD 700-MHz spectrometer equipped with a cryogenically cooled three-channel TCI probe and analyzed using MestReNova.

### 2.6 GPC

For the aqueous MPC homopolymer polymer, *M*_n_, *M*_w_, and dispersity were acquired using a Shimadzu Prominence-i LC-2030C 3D liquid chromatograph equipped with Shodex SB-803 HQ & SB-804 HQ columns (Showa Denko America) and RI and PDA detector. 1–2 mg of MPC homopolymers were mixed in 1 mL of 0.1 M NaNO_3_ water and passed through a 0.2 μm filter. Aqueous mobile phase on the columns was 0.1 M NaNO_3_ water. Columns were calibrated with PEG standards 0.575–700 kDa (PEG calibration kit, Agilent Technologies).

For copolymers, *M*_n_, *M*_w_, and dispersity were acquired from a PSS SECurity GPC system using Agilent 1260 Infinity instrument technology. The GPC system was equipped with two PFG combination medium microcolumns with 7 μm particle size (4.6 mm × 250 mm, separation range 100–1.000.000 Da), a PFG combination medium precolumn with 7 μm particle size (4.6 mm × 30 mm), and a refractive index (RI) detector. Mobile-phase composition was made of distilled HFIP with 0.019% sodium trifluoroacetate and run at 40 °C, with a 0.3 mL min^–1^ flow rate. Calibration was made with poly(methyl methacrylate) standards from PSS. Typically, 1–2 mg of polymer was dissolved in HFIP overnight and samples were filtered with a PTFE syringe filter.

Analysis of the *M*_n_, *M*_w_, and dispersity was completed with LabSolutions software (Shimadzu). GPC traces are found in Figure S3.

### 2.7 Infrared Spectroscopy

Infrared (IR) spectra (750–4000 cm^–1^) were collected for dried UPyMA (**2**) and copolymers (**3**) (0, 1.25, 2.5, and 5 mol %). The ATR mode on a Thermo Scientific spectrophotometer (Nicolet iS50 FTIR) was used and 32 scans were performed with background correction. Samples were tested as dried powders.

### 2.8 Thermal Analysis

Thermal characterization was performed on dry samples using a TA Instruments 250 differential scanning calorimeter (DSC). Radicalysed copolymers **4** were used for thermal analysis because **2** would lose mass during thermolysis of its end groups. Samples of **4** were then placed in a vacuum oven at 100 °C for one week to ensure all water was removed. Approximately 7–9 mg of sample was placed in an aluminum DSC pan and subjected to a heat/cool/heat/cool cycle over the temperature range of −80–160 °C at a linear heating rate of 10 °C min^–1^. The glass-transition temperature was determined from the second heating curve (DSC = Figure S9) and analyzed using TRIOS software (TA Instruments).

### 2.9 Dip-Coating Method

The base material, UPyE, was first dissolved in chloroform at 100 mg mL^–1^ and solvent cast (200 μL) on glass slides. The material was dried overnight under vacuum. Stock solutions of 50 mg mL^–1^ polymer **4** at different UPy ratios (0, 1.25, 2.5, and 5 mol %) were prepared by dissolving in HFIP. HFIP was chosen as the solvent for the coatings because it is known to interrupt UPy dimerization which we hypothesized would help facilitate cross coating adhesion.^37^ The bulk materials were dip coated into coating solutions of **4** for 1–2 s. The materials were left to dry in vacuum for one day.

### 2.10 Water Contact Angle

Hydrophilicity of the substrates after dip-coating with **4** was assessed using a drop-shape analyzer (Krüss DSA25 system). DSA4 software was used to calculate the water contact angle. 1 droplet was measured per material (*n* = 3). After 5 s of droplet’s contact with surface, photographs were taken and analysis was performed.

### 2.11 Challenging of Coatings

After drying the dip-coated materials, we challenged the coating using the following washing protocol. First, coated materials were submerged in PBS and then sonicated for 2 min at RT. Afterward, the samples were removed from PBS and washed twice with DI water and left to air dry in a Petri dish for 24 h before further testing.

### 2.12 Staining with Rhodamine 6G

For staining, the previously challenged substrates were immersed for 30 s in an aqueous solution of rhodamine 6G (0.02%) as previously described.^38^ They were washed with DI water three times and left to dry overnight at RT. Images of stained substrates were taken by fluorescence microscopy and assessed qualitatively as well as quantitatively, where the images’ mean gray value was obtained by ImageJ and normalized to the bare material.

### 2.13 BSA Absorption

Challenged substrates were submerged in a 0.01 mg mL^–1^ solution of Alexa-Fluor-488-labeled BSA in PBS for 24 hours at RT. The materials were then rinsed 5× with 4 mL of 10 mM PBS solution as previously described.^39^ After drying with nitrogen, the coatings were analyzed by fluorescence microscopy at 10× magnification. Settings were determined based on the positive control (bare UPyE without antifouling coating) and kept the same for the series of coatings. Image analysis was done to quantify the absorbance intensity. The materials tested did not exhibit background autofluorescence in the 488 nm channel. Therefore, using ImageJ software, the amount of BSA absorbed was compared easily by calculating the mean gray value of the images. The average of mean gray values (*n* = 3) was normalized to the average of the positive control (bare UPyE with no antifouling coating).

### 2.14 Cytotoxicity

A cytotoxicity test was performed using mouse embryonic fibroblasts (NIH-3T3). All cells were used at passages 4–6. The 3T3 cells were seeded at a density of 1 × 10^4^ cells per well on uncoated 24-well polycarbonate culture plates (Sigma-Aldrich) coupled with 12 mm Transwell with 0.4 μm Pore Polycarbonate Membrane Insert (Corning) for 24 h under 5% CO_2_ in a humidified incubator at 37 °C. After 24 h, the materials were placed inside the inserts and were thereby in indirect contact with the cells.

The cytotoxic effect of the coated material heterotelechelic copolymers 3 with cells was assessed in vitro using trypan blue (0.4%) (Thermo Fischer Scientific) dye exclusion. After 24 and 48 h, cells were dissociated into a single-cell suspension using trypsin-EDTA (0.05%) and phenol red (Thermo Fischer Scientific). The cell suspension was added to 1:1 (vol/vol) of FBS and centrifuged at 300*g*. The supernatant was then discarded and the pellet was resuspended in 1 mL of DPBS with 10% BSA. 10 μL of the cell suspension was mixed 1:1 with the trypan blue solution and the percentage of live cells was quantified using a TC20 automated cell counter (Bio-Rad).

### 2.15 Cell Adhesion

UPyE and coated materials (*n* = 3) were prepared in a 48-well plate, challenged as previously described, dried overnight, and sterilized by UV for 10 min. To increase cell attachment, the materials were incubated with FBS overnight. Mouse 3T3 fibroblasts were seeded on material surfaces to determine cell adhesion. First, cells were subcultured to 80–90% confluency in culture medium (DMEM and 10% FBS) in a T-175 flask. PBS was washed over the cells and 3 mL of trypsin/EDTA for 3–5 mins (37 °C) was used to detach the cells. Then, cells were diluted to 7 mL in culture medium and centrifuged for 5 mins at 0.5 rcf. After resuspension in 7 mL of medium, cells were counted and diluted to 500 000 cells/mL. FBS was removed from the materials, and 100 μL of cell suspension was seeded on each surface and left to attach for 1 h at 37 °C. An additional 100 μL of medium was added and cells were cultured for 24 and 48 h.

Before staining, cells were fixed with 4% formaldehyde in PBS for 30 min and subsequently permeabilized with 0.5% Triton X-100 in PBS for 15 min. After washing with PBS, Alexa Fluor 488 phalloidin (0.033 μM) was added to the samples and incubated at RT for 1 h. Phalloidin was removed with three washes of PBS, and DAPI (0.35 μg mol^–1^) was incubated with the samples for 5 min. The samples were all washed with PBS 3× and stored in PBS for imaging with an inverted fluorescence microscope. DAPI-positive nuclei were counted (five randomized areas photographed at 20× magnification of three substrates for each experiment) to give the cell count. The percentage adhered cells was calculated with eq 1 by dividing the cell count on coated surfaces by the average number of cells on the bare material
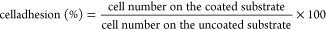
1

### 2.16 Statistical Analysis

Mean, standard deviation, and standard error of the mean (SEM) are presented. GraphPad Prism software was used to compare groups with one-way ANOVA with Tukey’s *post hoc* multiple comparison test where statistical significance was specified for *p* ≤ 0.05 unless stated otherwise.

## 3 Results

### 3.1 Synthesis

In our approach, we chose to use controlled radical polymerization to create well-defined polymers with different amounts of UPy incorporated into the copolymer backbone. To our knowledge, UPy (with hexamethylene spacers) incorporation into controlled radical polymers for antifouling coatings has been accomplished previously by its incorporation into the CTA or chain initiator.^40,41^ We envisioned creating a library of zwitterionic polymers in which a desired amount of UPy is decorated randomly along the chain of a controlled radical polymer. To impart UPy side chains into copolymers of MPC, we first synthesized a methacrylate monomer with iso-UPy (**1** in Scheme 1) according to the literature.^27^**1** was mixed with monomer HEMA and catalyst dibutyltin dilaurate in dry chloroform and heated to 90 °C. The UPy methacrylate (UPyMA; compound **2**) product was collected, purified, and confirmed by ^1^H NMR (Figure 2a; for detailed spectra in CDCl_3_ see Figure S1).

We polymerized copolymers of MPC and **2** by running the polymerizations with different mole percentages (0, 1.25, 2.5, and 5%) of UPyMA in the feedstock. Accordingly, we copolymerized MPC using chain-transfer agent (CTA), 4-cyano pentanoic acid dithiobenzoate, and ACVA thermal initiator to create a library of copolymers (Table 1). To showcase the targeting of different molecular weights, we adjusted the ratios from 100:0.5:0.1 (monomer/chain-transfer agent/initiator molar ratio) to 100:10:1 which enabled polymers with low molecular weight around 5000 g mol^–1^ to be synthesized (Figure S6 and Table S1). Because the RAFT technique offers good control over molecular weight, we prepared high-molecular-weight polymers (>10 000 g mol^–1^) to ensure **2** was added into the main chain. The CTA we chose to use in this reaction provided a heterotelechelic copolymer with a cyano pentanoic acid end group at the α-side and a dithiobenzoate end group at the ω-side of polymer **3**, with unimodal molecular-weight distribution (Figure S3). Of note, we also attempted the RAFT polymerization with a homotelechelic CTA to warrant a diacid with no need for end-group lysis; however, despite multiple changes in conditions, we consistently observed a bimodal distribution in molecular weight (Figure S7) which suggested poor control of polymerization. To render the homotelechelic copolymer **4**, we performed a radicalysis, as has been described for similar RAFT polymers.^42,43^ There is some concern in the literature that hydrolysis of the dithiobenzoate group could lead to cytotoxicity. Aminolysis of similar copolymers reduced toxicity by cleaving or capping the end groups. By utilizing radicalysis, the dithiobenzoate group could also be effectively removed, mitigating the risk of cytotoxicity and ensuring a safe interaction between cells and the material.^44^ In addition, removing the dithiobenzoate discolors the polymers, prevents malodor, and resolves the issue of background fluorescence during BSA absorption tests and cell fluorescence imaging, allowing for more accurate and reliable data collection. Aminolysis was first explored with 17 equiv of butylamine to CTA on a polymer, but after purification, we found that only 3% of end groups had been lysed. With radicalysis, however, we found the complete reduction of end groups after 4 h and chose this method for all subsequent polymers.

For the nomenclature of these polymers, we specify the mole percentage of UPy, such as MPC-UPy*_x_*, where *x* is mole percentage of the feed. To obtain ^1^H NMR spectra of the homopolymer (Figure 2b) and MPC-UPy_5_ (Figure 2c), we used a ratio of 90:10 chloroform-*d* and methanol-*d*_4_, respectively. The copolymers had limited solubility in most deuterated solvents and this particular ratio provided the best dissolution. Because UPy contains acidic exchangeable protons, the characteristic hydrogen-bonding amines are not visible in the methanol solvent; however, conveniently the aromatic proton at 5.8 ppm (proton labeled e’ in Figure 2c) allowed determination of the mole percentage of UPy on the backbone. Labeled spectra of MPC, MPC-UPy_1.25_, MPC-UPy_2.5_, and MPC-UPy_5_ can be found in Figures S2 and S4, as well as the comparative NMRs post radicalysis.

**Figure 3:**
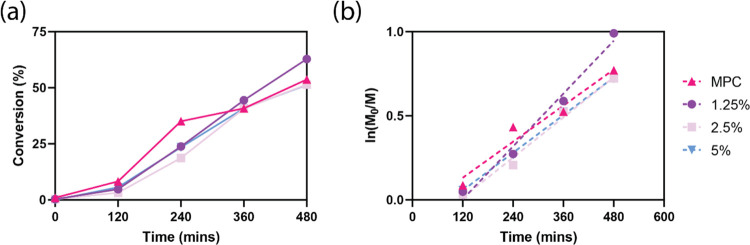
(a) Conversion percentage and (b) ln(*M*_o_/*M*) comparing RAFT polymerization of MPC (pink), 1.25 (purple), 2.5 (light purple), and 5 mol % UPy (blue) over the reaction time. *R*^2^ of MPC (0.9555), 1.25% (0.9840), 2.5% (0.9879), and 5 mol % (0.9974) shows fit of linear regression (dotted line) and supports linear first-order kinetics characteristic of RAFT polymerization, additional linear regression data available in Table S2. The monomer/chain-transfer agent/initiator molar ratio was 100:0.5:0.1.

Since there are only a few reports of UPy monomer polymerization with RAFT,^29,45,46^ we checked the linearity of the polymerization kinetics during the process. UPy-functionalized CTAs have already been shown to maintain the linear growth of RAFT polymers^41^ and thus a methacrylated version was expected to perform similarly. During the polymerization process, the chain growth was monitored by sampling at different reaction times (0, 120, 240, 360, and 480 min). ^1^H NMR was performed on the crude reaction mixtures, and the monomer concentration was used to calculate polymer conversion percentages (Figure 3a). A kinetic plot (Figure 3b) of ln(*[M*_0_]/[*M]*) shows a linear increase in molecular weight for polymerization of **3**, where [M] is the monomer concentration and [M_0_] is the initial monomer concentration, thus confirming our hypothesis that the UPy monomer could participate in characteristic RAFT polymerizations with pseudo-first-order kinetics.^47^ Under these reaction conditions, we found it difficult to incorporate more than 5 mol % UPy into the feedstock, as the copolymer (**3**) tended to precipitate out during the reaction when left for a similar reaction time (480 min).

The synthesized polymers were characterized with respect to UPy content (mol % and UPy/chain; Table 1) by ^1^H NMR of the purified product. Interestingly, the mol % UPy measured on the purified polymer corresponded to the feed ratio and % conversion; however, the polymerization had not gone to completion (it was typically stopped after 50%, which suggests the reactivity might be higher for the UPy monomer). With ^1^H NMR, however, straightforward mole percentage of monomer to polymer was verifiable and controllable molecular weights via RAFT synthesis were achieved. Next, we compared the copolymers’ molecular weights (theoretical = ∼56 000 g mol^–1^) by end-group analysis on ^1^H NMR. The molecular-weight distribution was found via GPC and compared to NMR in Table 1. MPC homopolymers were readily soluble in water and thus an aqueous eluent could be used; however, HFIP as an eluent had to be used for the remaining copolymers (Figure S3). Molecular weight calculated by NMR matched more closely to the theoretical *M*_n_ as opposed to *M*_n_ determination by chromatography. For example, for the MPC homopolymer at 54% conversion, we expected the *M*_n_ to be around 30 000 g mol^–1^, but by ^1^H NMR, we calculated 35 000 g mol^–1^; GPC, on the other hand, recorded a somewhat lower *M*_n_ of 21 000 g mol^–1^ (in H_2_O). However, when switching to the mobile phase of HFIP (needed to solubilize UPy-containing polymers), we noticed an increase in *M*_n_ to nearly 60 000 g mol^–1^. This increased molecular weight (via HFIP GPC) was also observed for the MPC-UPy_1.25_ sample, yet further increases in the amount of UPy led to lower recorded *M*_n_ in HFIP in realignment with the NMR data (Table 1). This shift in *M*_n_ as a function of UPy content is reminiscent of single-chain nanoparticles;^48^ however, further exploration is outside the scope of this study. Nevertheless, dispersity (*Đ*) by GPC demonstrated good control over molecular weight for all polymers synthesized in the library, with no discernable trends with increasing amounts of feed **2** and further no evidence of any covalent branching of the polymer **3** because of the unimodal distribution. After radicalysis to produce the copolymer **4**, the aromatic protons disappeared (Figure S4) and the dried material changed color (Figure S8) from pink to white, a classic indication of successful end-group cleavage. GPC *M*_n_ (mobile phase: HFIP; traces can be found in Figure S3) was performed on **4** and we found that radicalysis did not affect molecular weight and was thus a safe and effective alternative to aminolysis. Because end groups on **3** could be cleaved by thermolysis on a DSC, we used **4** without lysable end groups to determine the glass-transition temperatures *T*_g_ (Figure S9). Homopolymer MPC had a *T*_g_ at 25 °C and with the addition of UPy into the copolymer at small concentrations (1.25 and 2.5 mol %), the *T*_g_ decreased. Bulky UPy groups could disrupt the formation of dipolar cross-links between the zwitterionic MPC groups as has been seen elsewhere^49^ with MPC copolymers, thereby reducing the *T*_g_. However, when enough UPy had been added onto the chain, 5 mol % UPy (4 UPy/chain), the *T*_g_ increased to 21 °C near the homopolymer’s original *T*_g_. Such an increase could be supported by the increased presence of UPy and their supramolecular interactions with each other.

### 3.2 Infrared Spectroscopy

**Figure 4:**
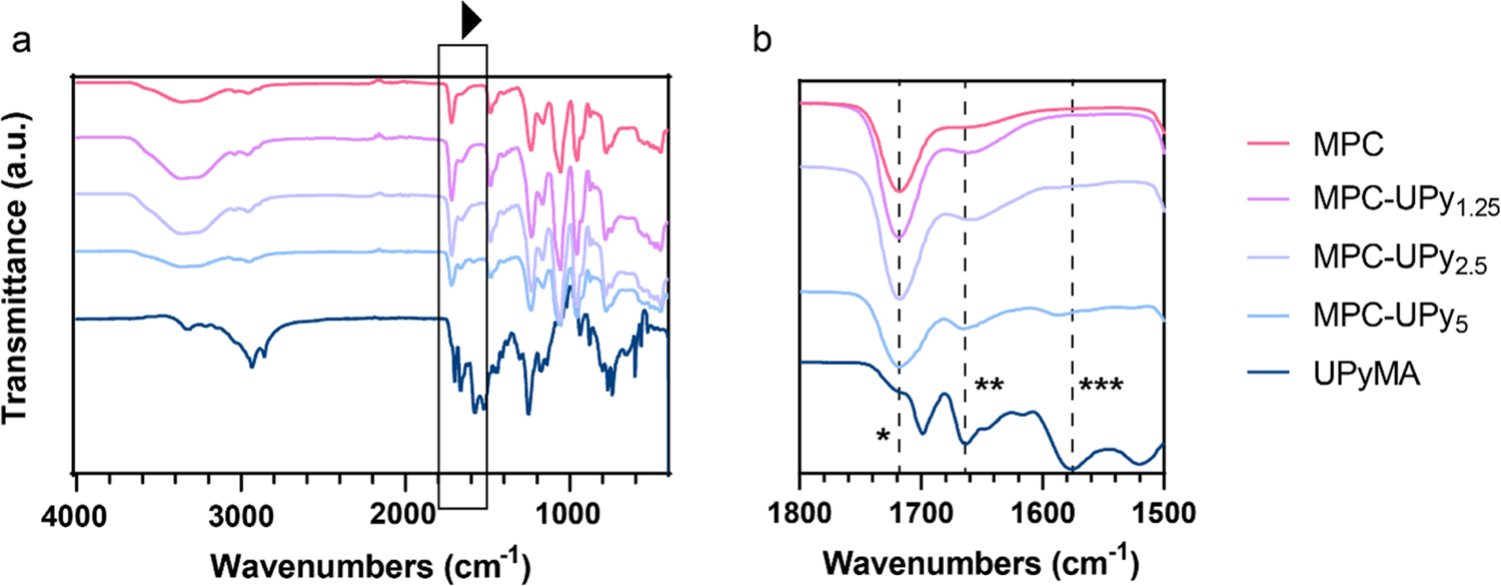
(a) FTIR spectra of dried MPC homopolymer (pink), 1.25 (purple), 2.5 (light purple), 5 mol % (blue) UPy, and UPyMA monomer (dark blue). (b) Zoomed in area of FTIR spectra between 1500 and 1800 cm^–1^ showcasing MPC’s carbonyl ester (1720 cm^–1^) and UPy’s urea carbonyl (∼1665 cm^–1^) and amide II band (∼1580 cm^–1^) shown as “*”, “**”, and “***”, respectively. Shift of amide II from UPyMA from 1580 to 1591 cm^–1^ in MPC-UPy_5_ supports UPy hydrogen bonding in MPC-UPy_5_. The curves have been off-set for clarity.

FTIR was performed to confirm UPy incorporation in the polymer and to probe the hydrogen-bonding interactions in this zwitterionic system. First, MPC could be detected by its carbonyl ester at 1720 cm^–1^ (Figure 4b, *), N^+^(CH_3_) stretching at 966 cm^–1^, and −POCH_2_ at 1084 cm^–1^. MPC peaks appeared to slightly decrease with increasing mol % of UPy because the polymer content of MPC decreased. Characteristic UPy peaks of the urea carbonyl (1667 cm^–1^, Figure 4b, **) and amide II band (1580 cm^–1^, Figure 4b, ***) appeared more defined in the 5 mol % sample; UPyMA monomer is shown for comparison. The amide II band, while negligible in the 1.25 and 2.5%, offered more insight into the hydrogen bonding of the UPy’s amide.^50^ In temperature studies by Appel,^51^ model UPy compounds showed that signals shift to lower wavenumbers when heat was applied. Heat has been used to disrupt UPy dimerization and increase processability. The hydrogen bonding therefore was disrupted which caused this particular amide II band to decrease. This gives us an indication on the hydrogen bonding of the system because this type of intermolecular force can affect the vibrational motion of the amide group. With this shift to higher wavenumbers (Figure 4b, ***, from 1580 to 1591 cm^–1^), we can detect that there was an increase in hydrogen-bonding in the 5 mol % polymer compared to other samples and UPyMA.^29^

### 3.3 Water Contact Angle

**Figure 5:**
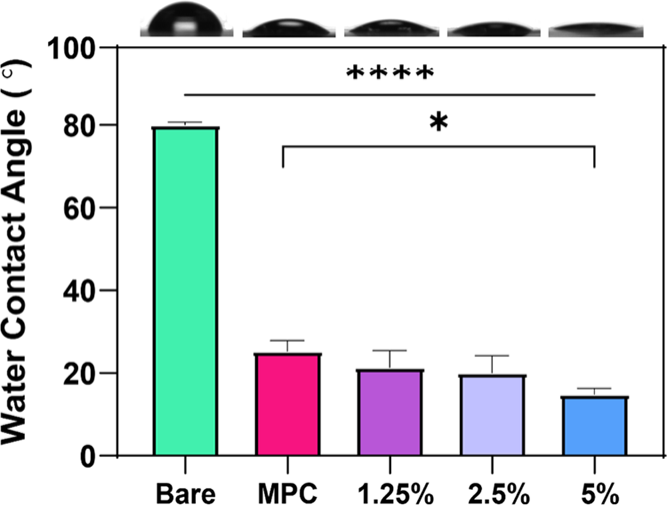
Water contact angles of bare material compared to dip-coated surfaces MPC homopolymer and 1.25, 2.5, and 5 mol %. Representative images are shown above each corresponding sample. Data expressed as mean with standard deviation (*n* = 3). ****indicates *p* ≤ 0.0001 and *indicates *p* ≤ 0.05 and no line drawn means there is no significant difference.

To test the ability of the zwitterionic coating to alter the surface properties of a base polymer, we used water contact angle for initial characterization on freshly coated surfaces (no challenge). A hydrophobic UPy-containing elastomer (UPyE) comprising a poly(hexamethylene carbonate) backbone chain extended with UPy moieties^34^ obtained from SupraPolix (Netherlands) was utilized as the base polymer, and all subsequent coatings were made via dip coating on glass from HFIP. Water contact angle was calculated after 5 s. A decrease in contact angle was immediately apparent (Figure 5) when coating the hydrophobic UPyE (80.0°) with the copolymers, MPC (25.2°), 1.25 mol % (21.4°), 2.5 mol % (20.1°), and 5 mol % (14.9°). Especially for the 5 mol % copolymer, the contact angle continued to decrease after the 5 s measurement point until completely wetting the surface. MPC coatings should produce a contact angle of about 20°, as was seen with the control; further, we saw a statistically significant decrease to 14.9° with 5 mol %. Others have proposed that decreased contact angle on their materials might be due to enhanced adhesion of the coating to the substrate’s surface.^13^

### 3.4 Coating of Polymers and Antifouling Experiments

To achieve a suitable surface for antifouling characterization, several coating processes were explored, including spin-coating, dip-coating, and drop-casting of our polymers on UPyE. Coating quality was improved with dip coatings according to visual analysis. For example, with spin-coated and drop-cast samples, the coatings were rough and opaque after drying, an indication of phase separation. Dip-coated samples, however, remained transparent and did not have visible signs of coating detachment. We proceeded with dip-coated samples to test coating quality and antifouling performance in more advanced studies. As the reservoir for dip coating, we created a solution of 50 mg mL^–1^ polymer in HFIP. The dip coating was performed on dry cast UPyE polymer, and after coating, the samples were allowed to dry under vacuum. With this procedure, we found that MPC homopolymer was also well coated on the surface of the elastomer. In order to test how well the coated UPy copolymers were adhered to the UPy elastomer, we created a methodology to challenge and remove loosely bound polymer. MPC homopolymer is superhydrophilic and, unless anchored on hydrophobic surfaces, the film will readily solubilize in water rendering the protein repellency impaired.^52^ Such a washing step would remove unbound MPC polymer from the surface and help us capture the effect that the UPy moieties had on preventing coating dissolution and maintaining antifouling performance. Thus, once materials were coated, we submerged them in PBS and immediately sonicated for 2 min to challenge the coating adhesion. The materials were then washed to remove unbound polymers and dried. We will refer to this protocol as the “challenging protocol”.

**Figure 6:**
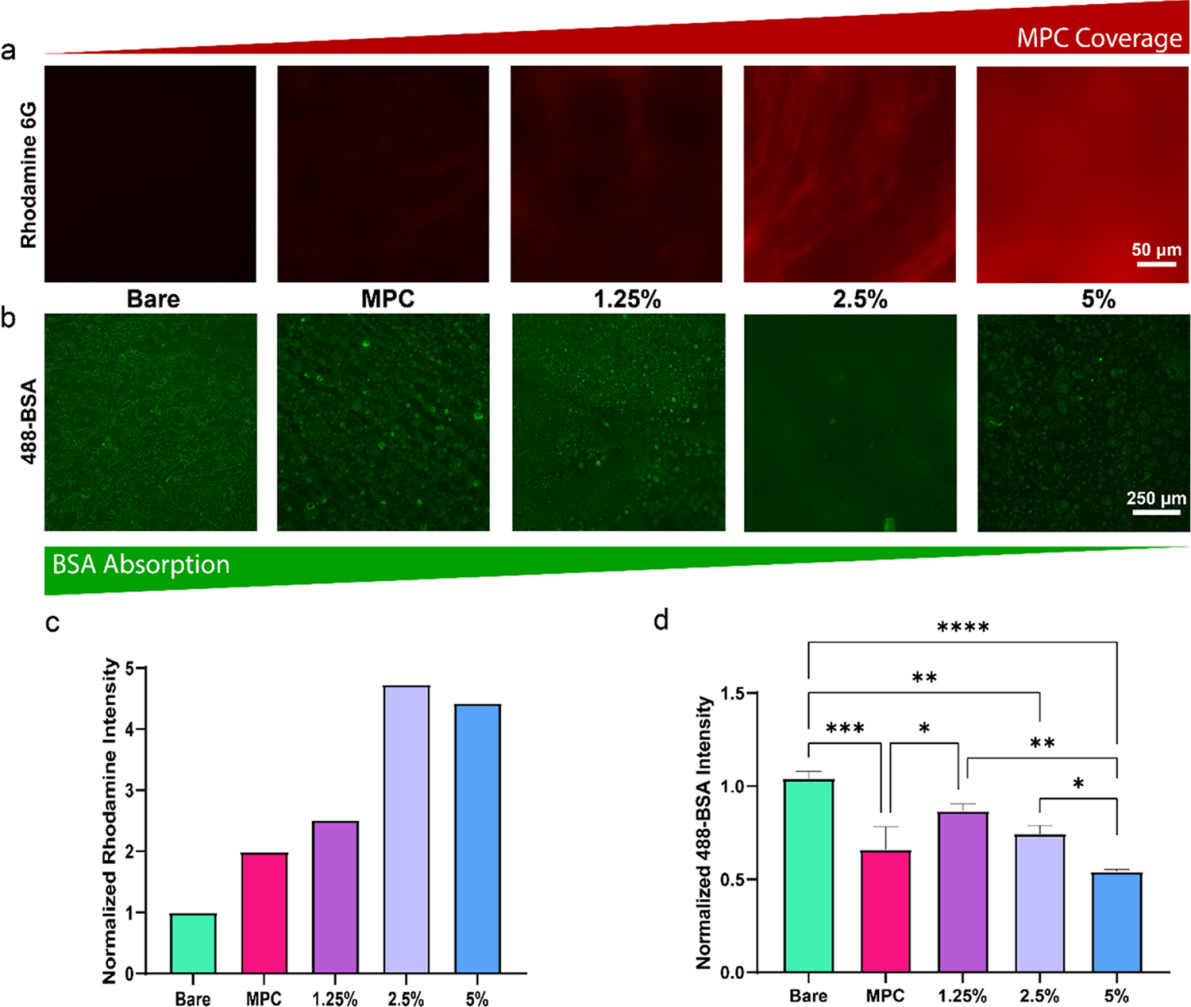
Coating quality and protein fouling study. (a) Fluorescence images after rhodamine 6G labeling of bare UPy elastomer, coated MPC, 1.25, 2.5, and 5 mol % UPy. Coating resilience increases with UPy %. Scale bar represents 50 μm. (b) Fluorescence images of materials after Alexa-488-conjugated BSA experiment. BSA antifouling was lowest with 5 mol % UPy. Scale bars represent 250 μm. (c) Quantification of the Rhodamine fluorescence. Data are normalized to the bare material. (d) Quantification of the BSA fluorescence. Data normalized to bare material fluorescence after BSA absorption and expressed as mean ± SD (*n* = 3). *indicates *p* ≤ 0.05, **indicates *p* ≤ 0.01, ***indicates *p* ≤ 0.001, ****indicates *p* ≤ 0.0001.

MPC coating quality was assessed by fluorescence imaging with rhodamine 6G. The association between rhodamine and phosphorylcholine has been used previously to judge the quality of medical device antifouling coatings.^53^ After our challenging protocol, we stained the materials by submerging in rhodamine solution. In Figure 6a, the rhodamine intensity is seen to increase with UPy concentration in the polymer, indicating an increase in MPC coverage. The fluorescence images of bare and coated MPC have little to no fluorescence, indicating few PC groups are present on the surface due to a lack of anchoring between the homopolymer and UPyE.

Antifouling experiments were performed on substrates challenged to remove any unbound polymer with Alexa-488-conjugated BSA. This biomacromolecule has been used as a model protein to assess surface fouling. BSA’s conjugation with a dye can be used to determine the affinity of the protein to a surface by fluorescence. Qualitative assessment of the BSA fluorescence (Figure 6b) across all samples indicated that 5 mol % had the least amount of fluorescence, especially with many areas showing no fluorescence at all. The 2.5 mol % sample appeared to have more fluorescent signal, homogeneously distributed over the sample. The 1.25 mol % sample, however, had increased prevalence of very bright spots of fluorescence or BSA, perhaps due to areas of high adsorption on the surface. To complete the series, we found that BSA covered the bare material’s surface in a more homogeneous fashion with more quantifiable fluorescence (Figure 6d) than with coated polymers of **4**. Taken together, the fluorescence signal decreased with increasing UPy mol %. We found significantly less fluorescence intensity with coated substrates, especially with 5 mol % (Figure 6d). This agree with our finding that MPC coating was immobilized with increased UPy.

### 3.5 Toxicity

**Figure 7:**
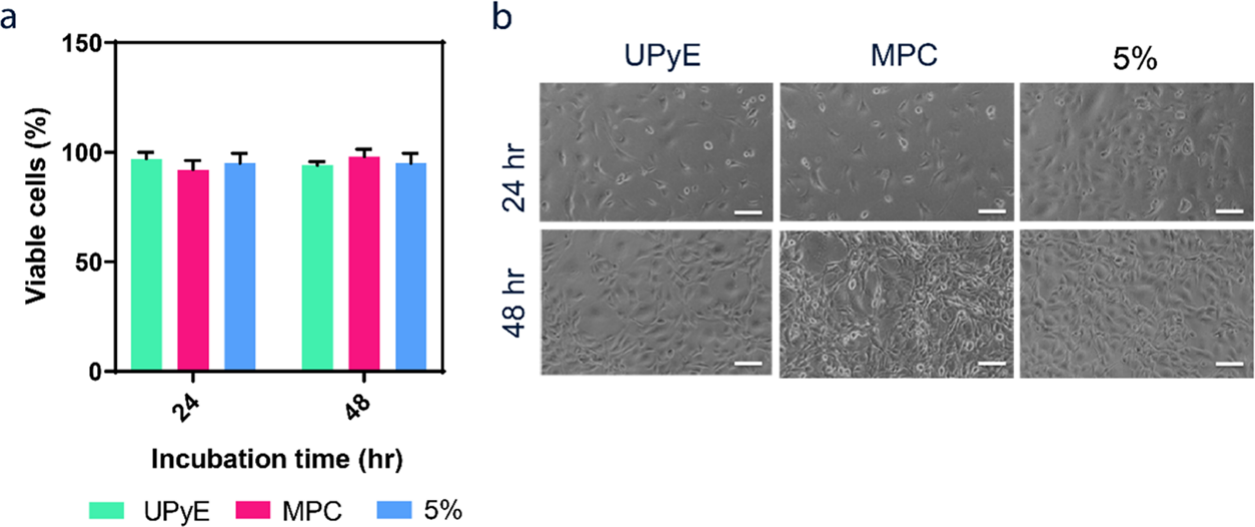
Toxicity screen of materials and coatings. (a) Viability of 3T3 cells incubated with the different coatings assessed by trypan blue staining, expressed relative to untreated cells (100% viability). Result are shown as means ± SEM of *n* = 3. (b) Brightfield pictures of 3T3 cells after 24 h (top row) and 48 h (bottom row) of culture in presence of UPyE (left column), MPC (middle column), and 5 mol % (right column). Scale bars represent 100 μm.

Both MPC’s and UPy’s cytocompatibility are well established in the literature,^54^ and the copolymerization of both MPC and **2** was expected to be well tolerated by cells. However, RAFT CTA’s dithiobenzoate has been suggested to result in possible hydrolysis and cytotoxic byproducts. To test this, we used **3** preradicalysis. Moreover, in order to confirm the nonadhesive nature of the materials, we should also confirm that the lack of adhered cells on coated materials was not simply due to cytotoxicity of the materials involved. Therefore, three materials were chosen for analysis based on our previous observations of coating quality and BSA antifouling: UPyE, homopolymer MPC, and copolymer **3** with 5 mol %, where 5 mol % resulted in the least amount of BSA absorbed and the most surface coverage after challenging. We analyzed 3T3 cell morphology and a viability assay in the presence of dip-coated substrates without washing or challenging to make sure the maximum amount of polymer was tested in the presence of cells. Of note, we used a noncontact cytocompatibiltiy test where the cells were adherent on cell culture plastic and not seeded on the materials. No significant difference in cell viability was observed for MPC and 5 mol % compared to UPyE, both after 24 and 48 h (Figure 7a). In addition, brightfield images showed that cells (Figure 7b) do not exhibit morphological differences when comparing coated and uncoated UPyE. Polymers of **3** are thus not cytotoxic, even if delaminated from the surface for low UPy % and MPC homopolymer.

### 3.6 Cell Adhesion

**Figure 8:**
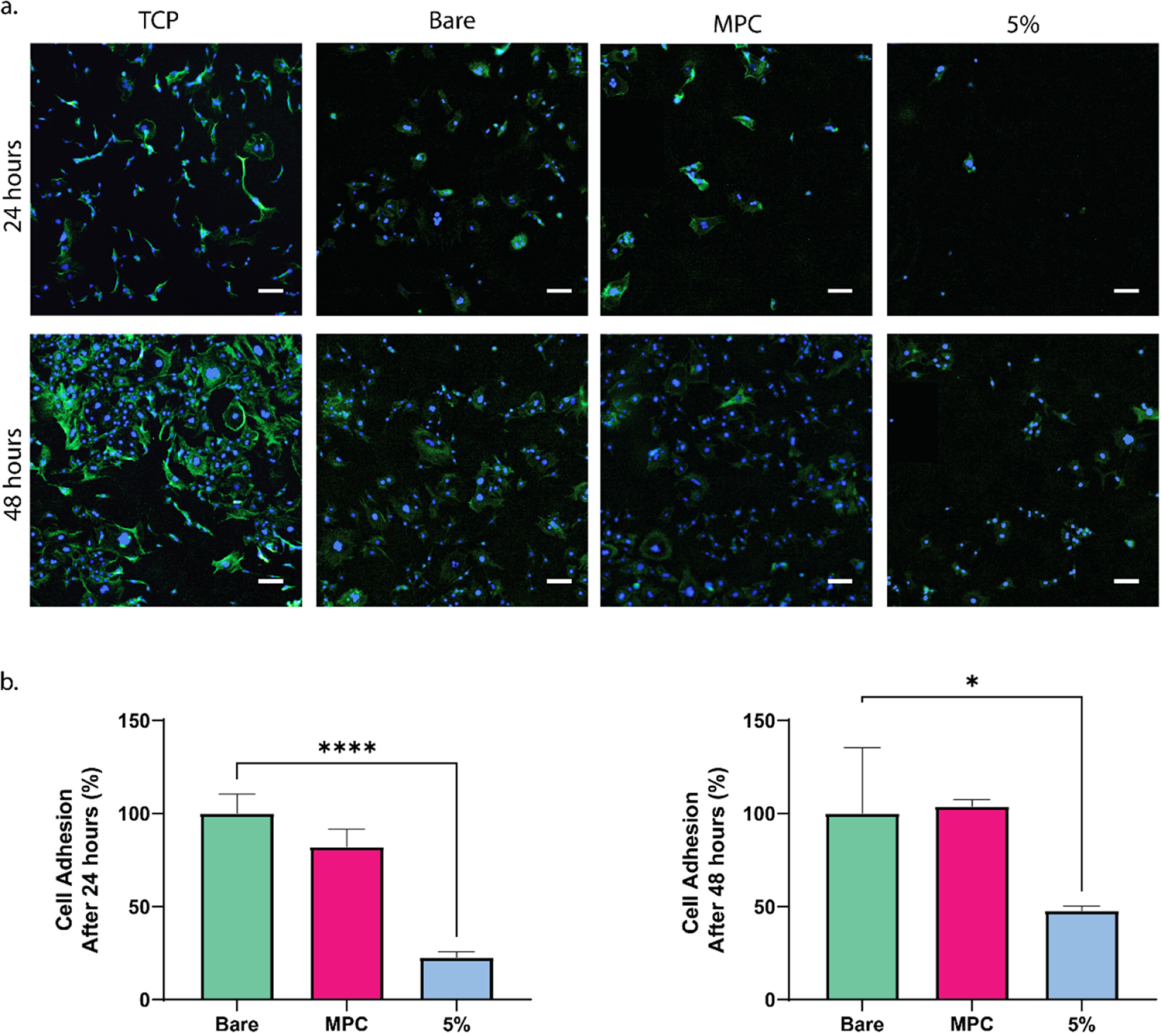
Cellular fouling of challenged surfaces of 3T3 cells stained for nuclei with DAPI (blue) and actin filaments with phalloidin (green). (a) Representative images of cellular fouling on TCP, bare UPyE, MPC-coated, and 5 mol % UPy indicating 78% less cells adhered to 5 mol % UPy copolymer compared to the bare material. The percentage 3T3 fibroblast adhesion compared to the bare material after (b) 24 h culture and after (c) 48 h. Scale bars represent 100 μm, UPyE, MPC, and 5 mol % expressed as mean ±SD (*n* = 3). *indicates *p* ≤ 0.05, **indicates *p* ≤ 0.01, ***indicates *p* ≤ 0.001, ****indicates *p* ≤ 0.0001, where no bar indicates no significant difference.

Antibiofouling can be evaluated in vitro by culturing cells on a coated substrate over a given time frame and counting the number of adhered cells on each surface compared to the uncoated substrate. The materials (UPyE, MPC, and 5 mol % UPy [copolymer **4**]) were treated with the challenging protocol so that unbound polymers would not interfere with cell attachment. Furthermore, by soaking the samples in FBS before cell seeding, we hoped to create a fouling environment more closely related to in vivo conditions, with complex protein compositions that could possibly adhere to the surface-enhancing cellular fouling. 3T3 mouse fibroblasts were seeded at 50 000 cells/cm^2^ onto the coatings. After 24 and 48 h, the cells were fixed and analyzed with DAPI and phalloidin staining (Figure 8a,b). Cell nuclei were counted and the percentage of adhered cells was quantified by dividing the number of cell nuclei on the coated material by the number counted on the noncoated material. (Figure 8b,c). For the 5 mol % UPy copolymer, the coating performed very well with a reduction of cell adhesion by 78% as compared to the noncoated elastomer. Similarly, other reports of copolymers of MPC were found to reduce cell adhesion by 50–80%.^38^ This is also consistent with our rhodamine quality assessment and BSA absorption tests in which the higher mol % of UPy improved coating quality and reduced protein fouling.

## 4 Discussion

Applying antifouling coatings to different material surfaces has been used to render medical devices not only resistant to protein adhesion and cell adhesion but also resistant to fibrotic capsule formation in vivo. Many of the anchoring mechanisms proposed in previous work employ covalent chemistry to immobilize zwitterionic coatings on surfaces. Some of these chemistries require multiple steps and can be substrate-dependent. Our work proposes a simple dip-coating process to immobilize coatings by utilizing a supramolecular pair: UPy–UPy dimerization.

This method offers greater flexibility than a grafting-from approach because of the clear characterization and control over the polymer structure. With the polymers presented herein, the synthesis of the supramolecular/zwitterionic polymers is precisely controlled in composition, i.e., molecular weight and functionality, i.e., mol % of UPy. This versatility enables tailoring polymer composition to meet coating requirements like surface hydrophilicity, protein repulsion, and cellular antifouling. Using presynthesized polymers makes it possible to take advantage of established polymerizations like RAFT and opens up the possibility to create tailor-made coatings via modular mixing before supramolecular attachment. Furthermore, “grafting to” in this manner minimizes the risk of altering polymer/device structure during the coating process. Because the polymer is synthesized and purified before coating, the risk of leaching contaminants or cytotoxic chemicals from the medical device is reduced. Lastly, in terms of manufacturability, this approach offers easier scalability and reproducibility than other methods like grafting from. These polymers can be produced easily at scale and thoroughly characterized before coating to the medical device; offering improved quality control, batch-to-batch consistency, and reproducibility of coating process.

We first created a library of zwitterionic MPC-UPy copolymers using RAFT polymerization. The purified polymers were found to incorporate the UPy as a side chain from the UPyMA monomer we used in a predictable manner. Evidence of UPy on the polymers was provided by first ^1^H NMR and subsequently by FTIR. We performed radicalysis on the polymers to remove the CTA which was shown to autofluoresce and could be a source of cytotoxicity.

The UPy elastomer was provided for us to try as a model substrate and infer antifouling performance. Dip coatings of the polymers on the elastomer were used to assess water contact angle and coating fidelity using rhodamine and 488-BSA assays. A washing step was included in our test methods to remove unbound polymers from the surface, which would help us distinguish if bound polymers were in fact anchored by the UPy group. We found that incorporation of UPy in the copolymers improved coating resistance to the washing protocol; more polymer was found anchored to the substrate, maintaining wetting properties (Figure S10) and improved BSA repulsion when mol % of UPy was above 2.5%. The increasing mol % of UPy afforded polymers that were immobilized on the surface and could functionalize the elastomer with antifouling properties. We selected 5 mol % to test for cytotoxicity and cellular antifouling. Even with unlysed CTA, RAFT copolymers were not found to exhibit changes in cell viability. This is good evidence that the decrease in cells in our adhesion studies was due to adhesion and not because they are unviable, and indeed the coatings were found to prevent cellular adhesion. Already, the bare material was not exceptionally cell adhesive but even under heavy fouling conditions (soaked in serum), we found the coating to have improved performance.

Interestingly, the coating seemed to work best within 24–48 h, after which cells start to adhere to the surfaces. The cells that do adhere to the coated surface appear to have shorter actin fibers and were less spread than on the control. After 48 h, more cells began to populate the coated surface, which could be due to the transitory nature of UPy–UPy interactions. A tunable, time-dependent coating could thus be designed by such a system. For example, where an implantable device is required to interact with host cells and not the immune system, a coating that cloaks the immune system from the material in the first days of implantation could then permit cell adhesion. The results of this study suggest that such a spatiotemporal coating could be designed when supramolecular UPy corresponds between substrate and a zwitterionic UPy copolymer.

In our study, we have discovered a crucial advantage associated with the use of directional supramolecular interactions for anchoring antifouling coatings: the ability to exploit a temporal response. Unlike covalent bonds, which are typically permanent or difficult to alter, supramolecular interactions offer a modular and functional approach that allows for tailoring the efficacy of the coating over time. By employing supramolecular interactions, we have developed coatings with dynamic properties. This temporal response mechanism opens up exciting possibilities for optimizing the antifouling performance of the coating in different environments and under varying conditions. The spontaneous disassembly property of supramolecular interactions enhances the reusability and sustainability of the coating, making it a promising pathway for the development of advanced antifouling strategies. We believe that exploring and understanding these benefits will contribute significantly to the field and open up new avenues for the design and application of supramolecular-based coatings.

Another significant advantage of utilizing directional supramolecular interactions lies in their spontaneous assembly and disassembly. Covalent bonds often require harsh conditions or specific chemical treatments to be formed and broken, which can limit the functional-group tolerance, processability, reusability, and versatility of the coating. In contrast, supramolecular interactions can be selectively and reversibly assembled and disassembled under milder conditions, facilitating the easy installation and removal, and provide pathways toward recycling of the coating when necessary.

## 5 Conclusions

In this study, we have shown the synthesis of copolymers using MPC and ureidopyrimidinone (UPy) monomers. RAFT polymerization was used to control molecular weight and functionalize MPC polymers with UPyMA. ^1^H NMR and FTIR confirmed copolymerization. Radicalysis was performed to remove end group and create homotelechelic polymers. After dip coating these polymers, we were able to characterize a protein-resistant surface on the supramolecular elastomer UPyE by water contact angle, a rhodamine 6G assay, and a BSA fluorescence assay. Via a 3T3 fibroblast cell line, we confirmed biocompatibility and the decrease and delay of cellular adhesion to the elastomer. Longer-term studies and time-course studies would be needed to explore the antibiofouling functionality over time. With these results, we show an increase in coating resilience and quality with a copolymer of 5 mol % UPy when compared to a MPC homopolymer. This particular composition can be used in further studies to test their ability to prevent foreign-body reaction.

## Supporting Information Available

NMR spectra of monomer UPyMA, copolymers 0 and 5%, and polymers before/after radicalysis steps; GPC traces of polymer; linear regression data of kinetics; second heating curve taken from DSC; water contact angle after dip coating and after challenging protocol; and properties of low-molecular-weight-targeted MPC homopolymer (PDF)
