# Supplementary material for: Complementary Supramolecular Functionalization Enhances Antifouling Surfaces: A Ureidopyrimidinone-Functionalized Phosphorylcholine Polymer
Source: ACS Biomater Sci Eng. 2023 Jul 6;9(8):4619–31. doi: 10.1021/acsbiomaterials.3c00425 (PMC10428092; doi:10.1021/acsbiomaterials.3c00425)
Supplement: Supplementary file 1 — ab3c00425_si_001.pdf [file ab3c00425_si_001.pdf]

Complementary Supramolecular Functionalization Enhances Antifouling Surfaces: A Ureidopyrimidinone Functionalized Phosphorylcholine Polymer

*Antonio J. Feliciano[1], Eduardo Soares[1], Anton W. Bosman[2], , Clemens van Blitterswijk[1], Lorenzo Moroni[1], Vanessa L.S. LaPointe[1], Matthew B. Baker[1,] **

1 Maastricht University, MERLN, Universiteitssingel 40, 6229 ER Maastricht, The Netherlands

2 SupraPolix B.V., Horsten 1, 5612 AX Eindhoven, The Netherlands

* correspondence can be addressed to m.baker@maastrichtuniversity.nl

Supplementary Information

Figure S1. ^{1}H NMR spectra of UPyMA

Figure S2. ^{1}H NMR spectra of MPC homopolymer (0%) and MPC-UPy5 (5%)

Figure S3. GPC traces of MPC, 1.25, 2.5, 5 mol%

Figure S4. ^{1}H NMR spectra and GPC traces before and after radicalysis

Figure S5. ^{1}H NMR spectra of copolymer library after radicalysis

Figure S6. GPC traces of low molecular weight MPC

Table S1. Properties of low molecular weight targeted MPC homopolymer

Figure S7. GPC traces of MPC homopolymers made with homo telechelic CTA

Table S2. Polymerization kinetics linear regression data

Figure S8. Color change of RAFT polymer before (left) and after (right) radicalysis.

Figure S9. Second heating curve taken from DSC

Figure S10. Water contact angle after dip coating and after challenging protocol

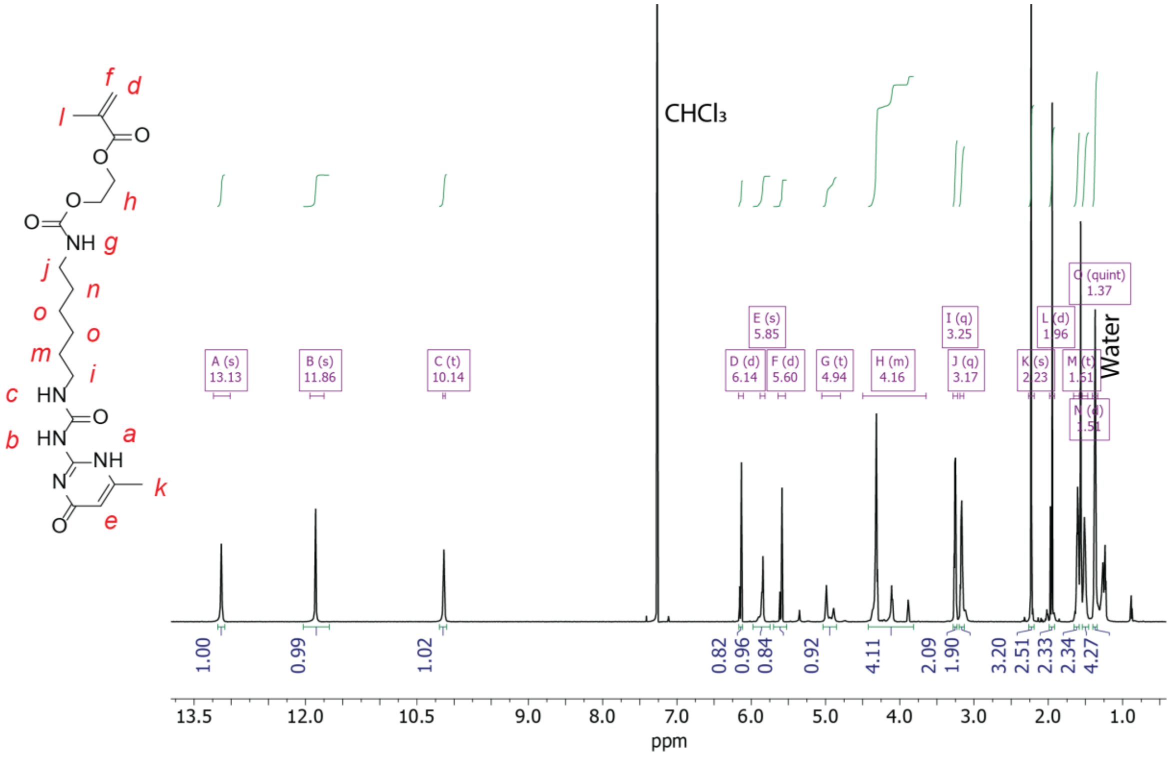

Figure S1. *^{1}H NMR spectra of UPyMA monomer **2** in $CDCl_3$.*

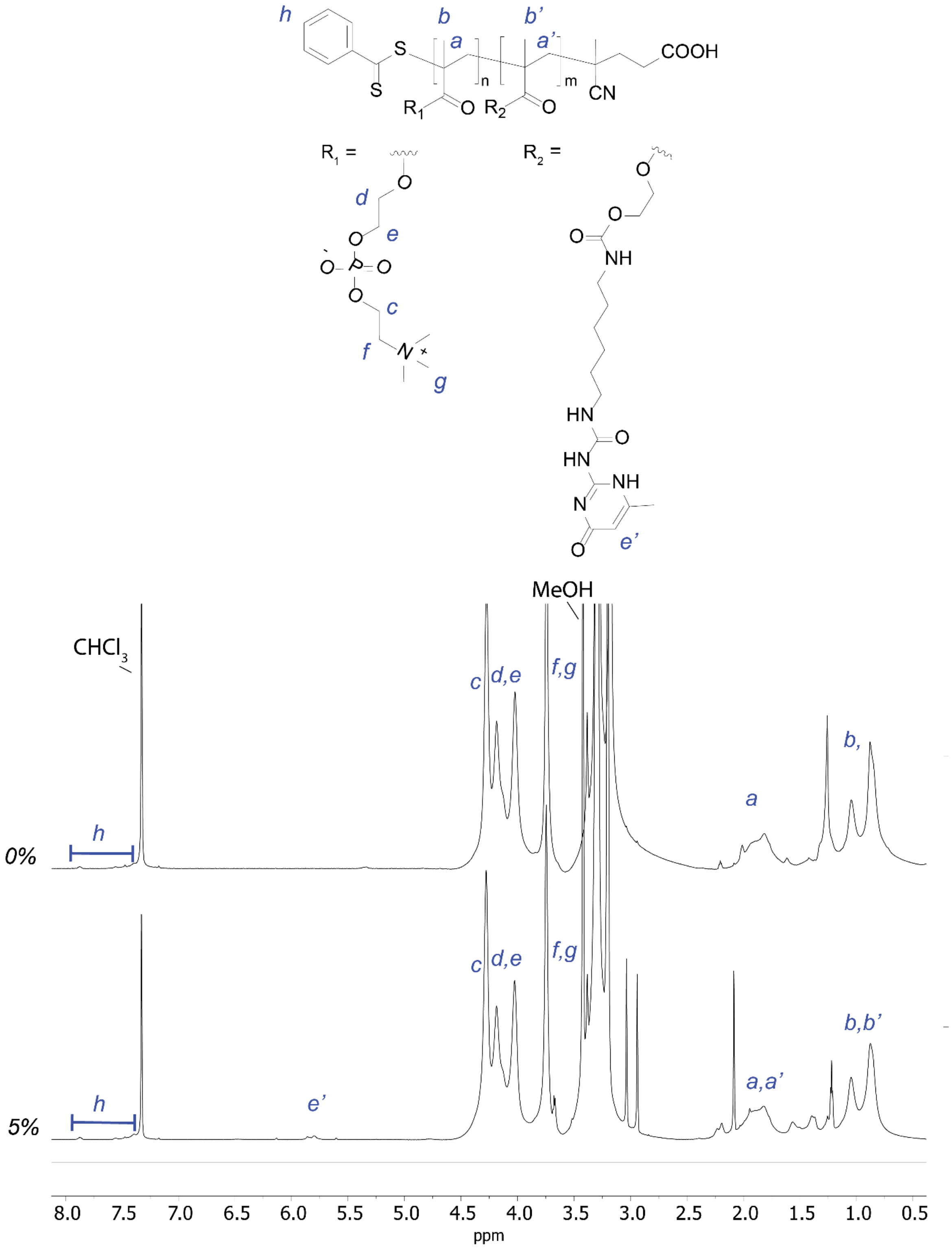

*Figure S2. Representative ^{1}H NMR spectra of MPC homopolymer (0%) and MPC-UPy$_5$ (5%) copolymer **3** in $CDCl_3$/MeOD (90:10).*

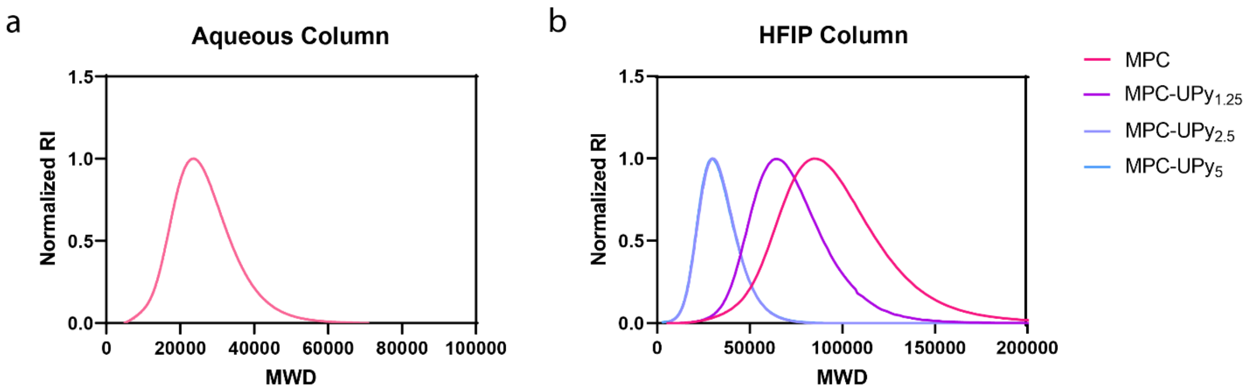

Figure S3. *GPC traces of* ***3*** *for MPC in (a) aqueous column and (b) 1.25, 2.5, 5 mol% in HFIP column, MWD (molecular weight distribution).*

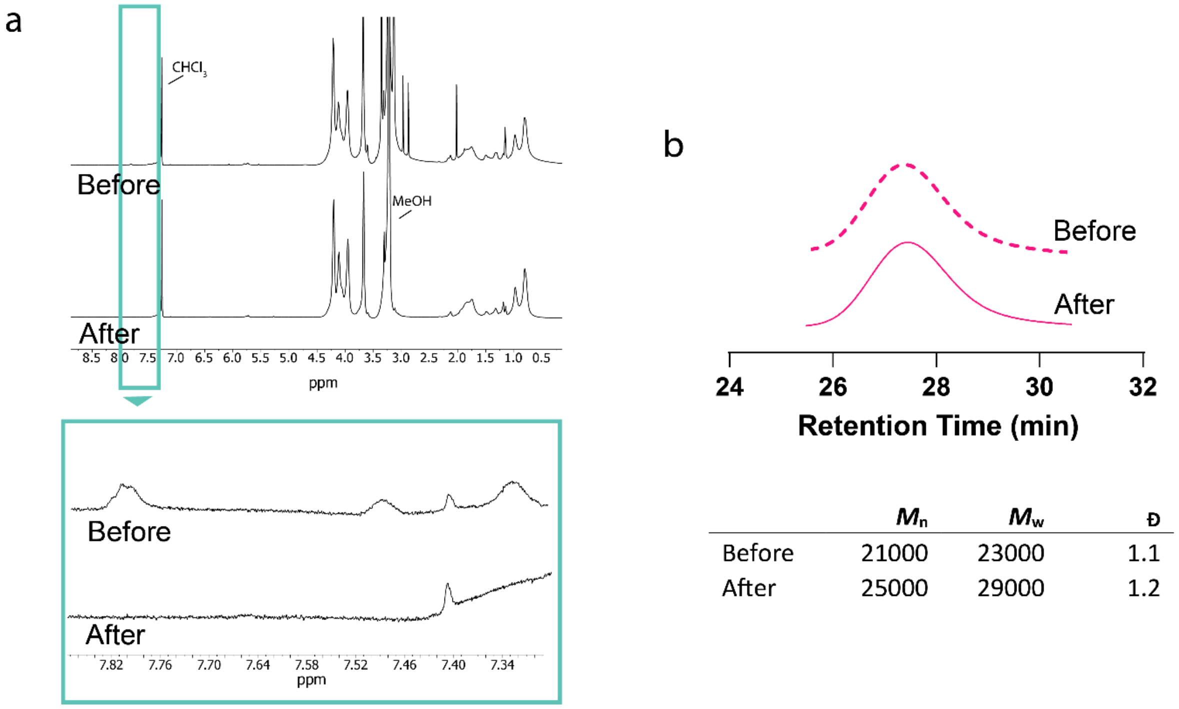

	M_n	M_w	Đ
Before	21000	23000	1.1
After	25000	29000	1.2

Figure S4. *^{1}H NMR spectra of* ***3*** *(5 mol%) (a) before and after radicalysis in $CDCl_3$/MeOD. Successful cleavage of CTA's aromatic protons post radicalysis. GPC of* ***3*** *before and after radicalysis,* ***4****.*

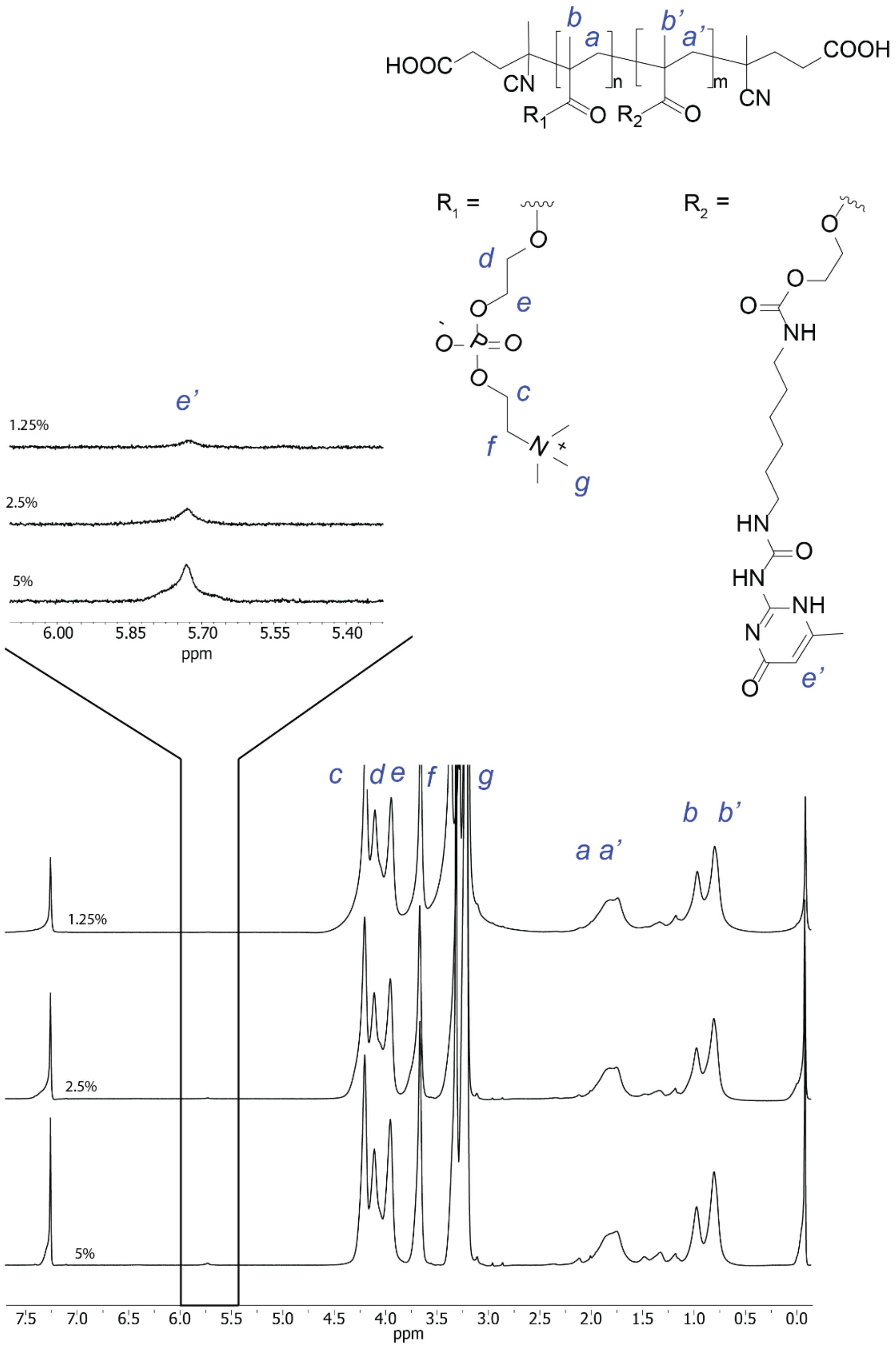

Figure S5. Representative ^{1}*H NMR spectra of copolymer library after radicalysis, copolymer* ***4****, with 1.25%, 2.5%, 5% UPy in* $CDCl_3$*/MeOD (90:10). e' proton in copolymers used to calculate mol %.*

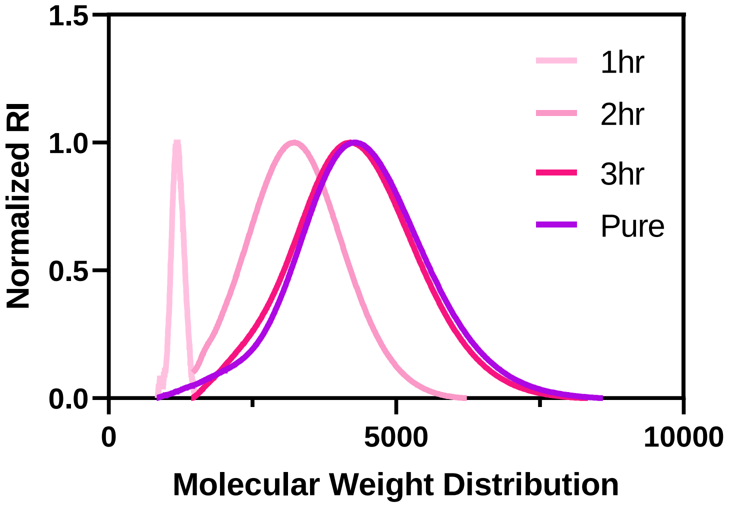

Figure S6. MWD of homopolymer MPC targeting lower molecular weights. The monomer: chain transfer agent: initiator molar ratio was 100:5.0:1.0.

Table S1. Properties of low molecular weight targeted MPC homopolymer

	^{1}H NMR	GPC$_{Aq}$		
**Reaction Step**	**M_n**	**M_w**	**M_n**	**Đ**
RAFT Polymerization	7100	4500	4700	1.1
Radicalysis	5600	4800	5100	1.1

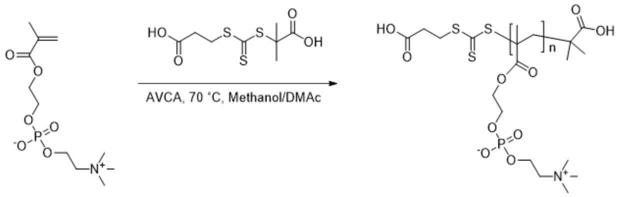

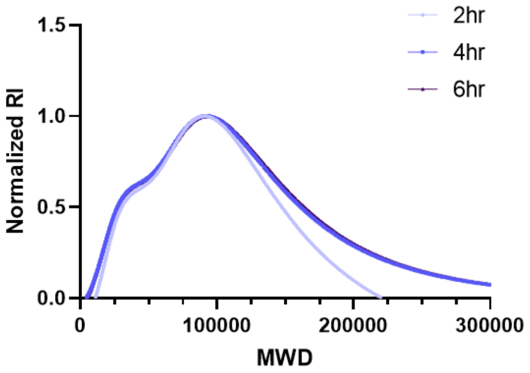

Figure S7. MWD of MPC homopolymers made with homo telechelic CTA 2-((((2-carboxyethyl)thio)carbonothioyl)thio)-2-methylpropanoic acid does not indicate successful RAFT polymerization due to broad bimodal distribution.

Table S2. Polymerization kinetics linear regression data.

Polymer 1	Slope	R^2
MPC	0.003372	0.9994
1.25	0.002618	0.9840
2.5	0.001988	0.9879
5	0.001867	0.9974

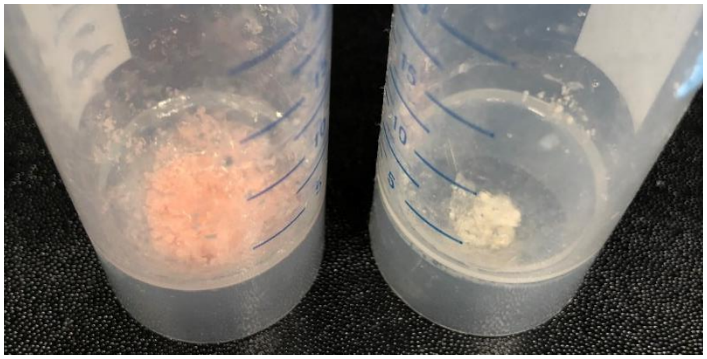

Figure S8. Color change of RAFT polymer before (left) and after (right) radicalysis.

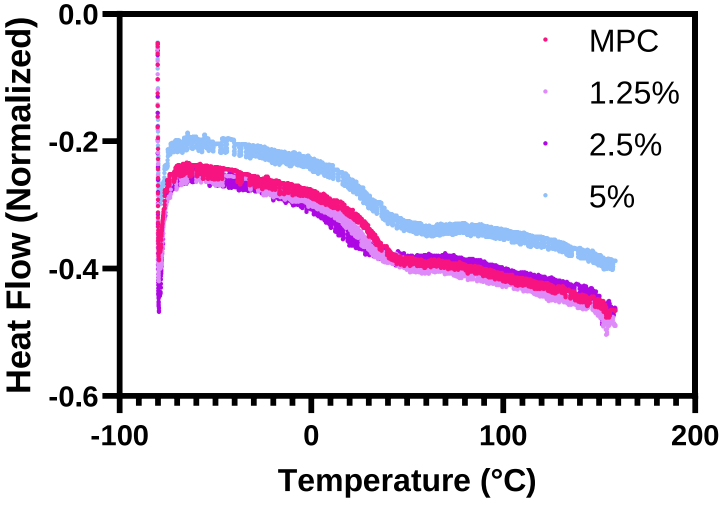

Figure S9. Second heating curve taken from DSC at linear heating rate of 10 °C min^{-1}*.*

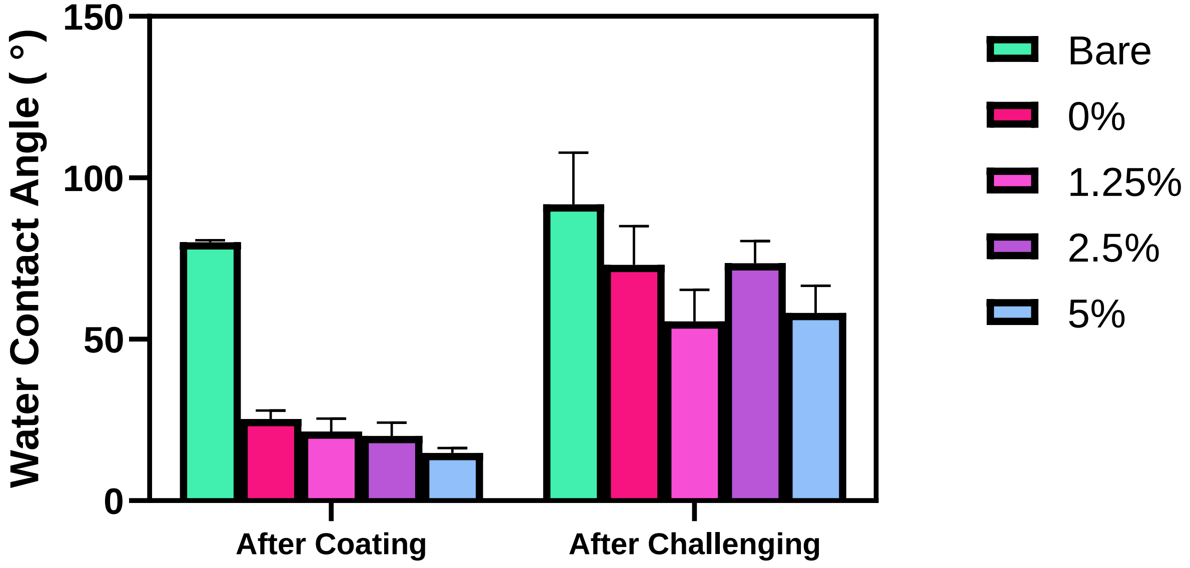

Figure S10. Water contact angle after dip coating and after challenging protocol. Increase in water contact angle suggests a decrease in coated MPC.
